# Supplementary material for: Interactions between immune cell types facilitate the evolution of immune traits
Source: Nature. 2024 Jun 12;632(8024):350–6. doi: 10.1038/s41586-024-07661-0 (PMC11306095; doi:10.1038/s41586-024-07661-0)
Supplement: Supplementary file 1 — This file contains Supplementary Fig. 1 and legends for Tables 1–11. [file 41586_2024_7661_MOESM1_ESM.pdf]

nature portfolio

https://doi.org/10.1038/s41586-024-07661-0

Supplementary information

Interactions between immune cell types facilitate the evolution of immune traits

In the format provided by the authors and unedited

Supplementary Information accompanying

Interactions between immune cell types facilitate the evolution of immune traits

Tania Dubovik[1,‡,*], Martin Lukačišin[1,*], Elina Starosvetsky[1,‡], Benjamin LeRoy[2,§], Rachelly Normand[1,¶], Yasmin Admon[1,‡], Ayelet Alpert[1], Yishai Ofran[1,3], Max G'Sell[2], Shai S. Shen-Orr[1,#]

[1] Department of Immunology, Faculty of Medicine, Technion - Israel Institute of Technology, Haifa, Israel
[2] Department of Statistics, Carnegie Mellon University, Pittsburgh, USA
[3] Department of Hematology and Bone Marrow Transplantation, Rambam Health Care Campus, Haifa, Israel.

* Co-first author
[#] Correspondence: shenorr@technion.ac.il
[‡] Present address: CytoReason, Tel-Aviv, 6701101, Israel
[§] Present address: Nike, Beaverton, OR 97005, USA
[¶] Present address: Broad Institute of MIT and Harvard, Cambridge, MA, 02142, USA

Supplementary Figures

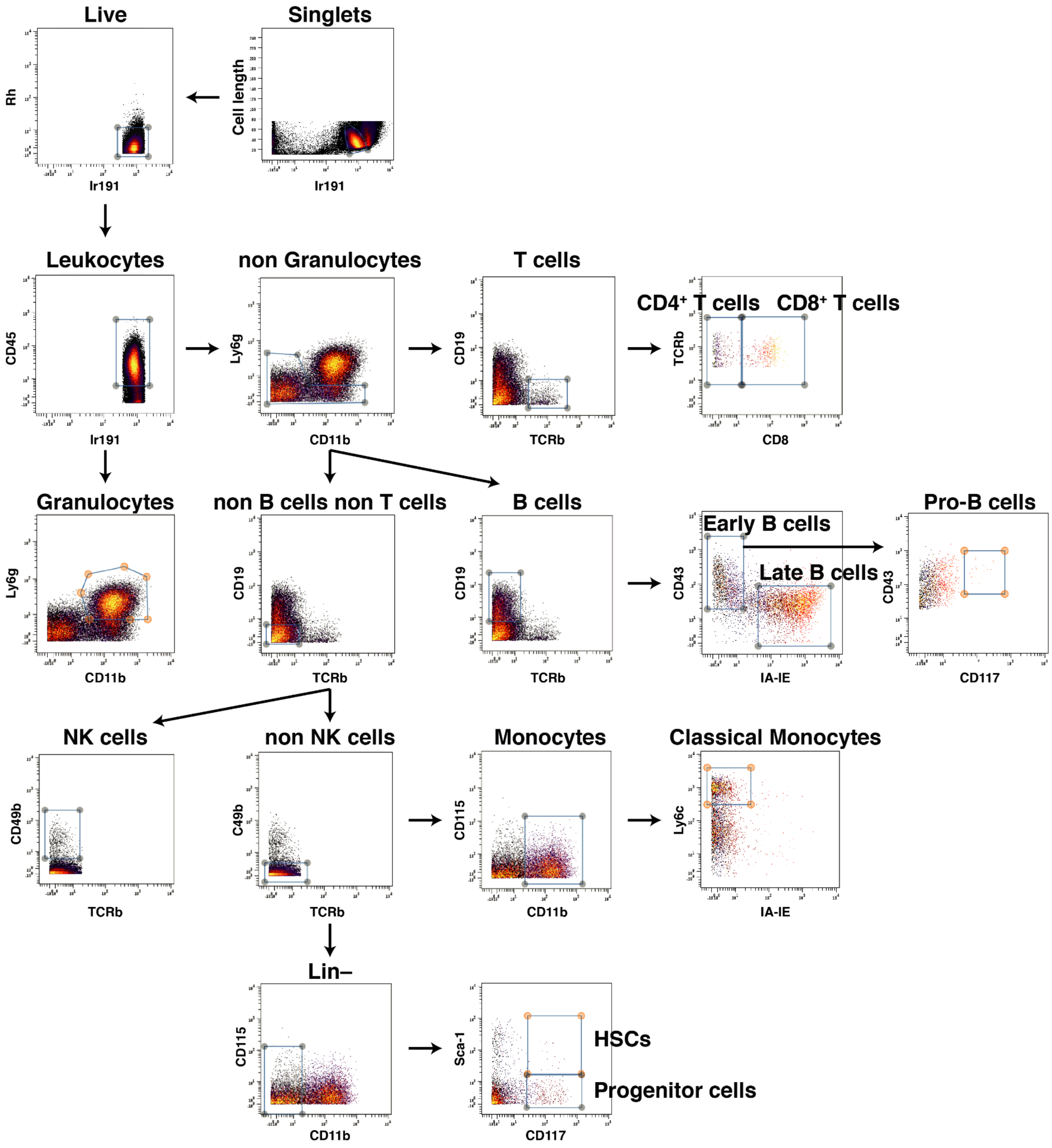

Supplementary Figure 1: Gating strategy for quantifying immune cell types in mouse bone marrow

CyTOF plots showing the gating strategy. Classical monocytes are referred to as ‘monocytes’ in the text. ‘Stem cells’ in the text correspond to a union of ’HSCs’ and ‘Progenitor cells’ subpopulations in this figure.

Supplementary Tables

Supplementary Table 1: Mouse strains used in this study

List of mouse strains used in this study. CC mice are listed using standard CC strain identifiers; founder strains using the full strain name. The column indicates in which cohort the mouse was profiled.

Supplementary Table 2: Antibodies used in this study

List of all markers and corresponding CyTOF antibodies that were used in this study, including clone, vendor and the cell-type it was used to identify.

Supplementary Table 3: Cell type markers detected in the exploratory cohort

List of immune cell subsets for each CC strain in the first cohort of mice which could be detected in that strain using our antibody panel. 1 = detected, 0 = not detected.

Supplementary Table 4: Immune profiles of the exploratory cohort

Frequencies for each cell type identified in bone marrow of CC mice of the first cohort. Frequencies are stated as a fraction of the given cell type over the total gated live cells.

Supplementary Table 5: Cell type markers detected in the validation cohort

List of immune cell subsets for each CC strain in the second cohort of mice which could be detected in that strain using our antibody panel. 1 = detected, 0 = not detected.

Supplementary Table 6: Immune profiles of the validation cohort

Frequencies for each cell type identified in bone marrow of CC mice of the first cohort. Frequencies are stated as a fraction of the given cell type over the total gated live cells.

Supplementary Table 7: Genetic associations determined

List of gene-cell type frequency associations determined in this study. The table additionally states whether the gene is associated in *cyto-cis* or *cyto-trans*, the average PhastCons conservation score of the associated gene, the associated polymorphism and whether the gene is associated with multiple cell types.

Supplementary Table 8: Enrichment analysis of associated genes

The results of gene enrichment analysis for genes associated with immune cell frequencies in mouse bone marrow performed using Ingenuity Pathway Analysis. This analysis was performed on a larger list of genes after relaxing the stringency of the association test (see *Methods*).

Supplementary Table 9: Functional enrichment analysis of *cyto-trans* genes

The results of gene enrichment analysis for genes associated with immune cell frequencies in mouse bone marrow in *cyto-trans*, performed using Ingenuity Pathway Analysis.

Supplementary Table 10: Mapping of Imm-Gen data to cell subtypes in this study

List of the ImmGen Consortium bone marrow samples for which the microarray gene expression data were used in this study, along with the annotation of respective cell subtypes to which these samples were assigned.

Supplementary Table 11: Genetic associations from human study used for evolutionary conservation analysis

List of gene-cell type frequency associations pooled from studies on human blood[28,29]. The table additionally states whether the association is in *cyto-cis* or *cyto-trans*, the average PhastCons conservation score of the associated gene, and the study in which the association was reported.
