# Supplementary material for: Interactions between immune cell types facilitate the evolution of immune traits
Source: Nature. 2024 Jun 12;632(8024):350–6. doi: 10.1038/s41586-024-07661-0 (PMC11306095; doi:10.1038/s41586-024-07661-0)
Supplement: Supplementary file 2 — Reporting Summary [file 41586_2024_7661_MOESM2_ESM.pdf]

nature portfolio

Corresponding author(s): Shai S. Shen-Orr

Last updated by author(s): May 8, 2024

Reporting Summary

Nature Portfolio wishes to improve the reproducibility of the work that we publish. This form provides structure for consistency and transparency in reporting. For further information on Nature Portfolio policies, see our Editorial Policies and the Editorial Policy Checklist.

Statistics

For all statistical analyses, confirm that the following items are present in the figure legend, table legend, main text, or Methods section.

n/a	Confirmed	
☐	☒	The exact sample size (*n*) for each experimental group/condition, given as a discrete number and unit of measurement
☐	☒	A statement on whether measurements were taken from distinct samples or whether the same sample was measured repeatedly
☐	☒	The statistical test(s) used AND whether they are one- or two-sided<br>*Only common tests should be described solely by name; describe more complex techniques in the Methods section.*
☐	☒	A description of all covariates tested
☐	☒	A description of any assumptions or corrections, such as tests of normality and adjustment for multiple comparisons
☐	☒	A full description of the statistical parameters including central tendency (e.g. means) or other basic estimates (e.g. regression coefficient) AND variation (e.g. standard deviation) or associated estimates of uncertainty (e.g. confidence intervals)
☐	☒	For null hypothesis testing, the test statistic (e.g. *F*, *t*, *r*) with confidence intervals, effect sizes, degrees of freedom and *P* value noted<br>*Give P values as exact values whenever suitable.*
☒	☐	For Bayesian analysis, information on the choice of priors and Markov chain Monte Carlo settings
☒	☐	For hierarchical and complex designs, identification of the appropriate level for tests and full reporting of outcomes
☒	☐	Estimates of effect sizes (e.g. Cohen's *d*, Pearson's *r*), indicating how they were calculated

Our web collection on statistics for biologists contains articles on many of the points above.

Software and code

Policy information about availability of computer code

Data collection	CyTOF 1 acquisition software (v1) was used to collect the raw data from CyTOF machine.
Data analysis	Cytobank (>= 3.2.1) was used to upload , explore and manually gate the fcs files.<br>Enrichment analysis was done using the IPA Spring release 2016 (Qiagen).<br>RStudio ,<br>Jupiter Notebook,<br>Custom script was used for all the analytical steps is available on Github : https://github.com/shenorrLabTRDF/CCanalysis

For manuscripts utilizing custom algorithms or software that are central to the research but not yet described in published literature, software must be made available to editors and reviewers. We strongly encourage code deposition in a community repository (e.g. GitHub). See the Nature Portfolio guidelines for submitting code & software for further information.

Data

Policy information about availability of data

All manuscripts must include a data availability statement. This statement should provide the following information, where applicable:

- Accession codes, unique identifiers, or web links for publicly available datasets
- A description of any restrictions on data availability
- For clinical datasets or third party data, please ensure that the statement adheres to our policy

Datasets generated in the study:
The raw fcs files are available on community.cytobank.org, experiment numbers 116506, 116507 :
Publicly available datasets that were used through the study:
ImmGen Microarray Phase 1 - GSE15907
Human Protein Atlas - https://www.proteinatlas.org/about/download
Conservation scores from UCSC Genome Browser :
https://genome.ucsc.edu/cgi-bin/hgTrackUi?db=mm10&g=cons60way
https://genome.ucsc.edu/cgi-bin/hgTrackUi?db=hg19&g=cons100way

Research involving human participants, their data, or biological material

Policy information about studies with human participants or human data. See also policy information about sex, gender (identity/presentation), and sexual orientation and race, ethnicity and racism.

Reporting on sex and gender	N/A
Reporting on race, ethnicity, or other socially relevant groupings	N/A
Population characteristics	N/A
Recruitment	N/A
Ethics oversight	N/A

Note that full information on the approval of the study protocol must also be provided in the manuscript.

Field-specific reporting

Please select the one below that is the best fit for your research. If you are not sure, read the appropriate sections before making your selection.

☒ Life sciences ☐ Behavioural & social sciences ☐ Ecological, evolutionary & environmental sciences

For a reference copy of the document with all sections, see nature.com/documents/nr-reporting-summary-flat.pdf

Life sciences study design

All studies must disclose on these points even when the disclosure is negative.

Sample size	No statistical calculations were done to predefine mice sample size. We have collected all the available CC strains at the UNC facility as of 2016. The CC mice were collected in duplicates to allow discovery of loci with smaller effect size.
Data exclusions	Exclusions were made based on data quality. In particular, low-viability bone marrow samples (by Countess and tripan staining) were excluded.
Replication	The CC mice were measured in duplicates, CC founder strains were measured in triplicates. We excluded replicates in case of the low cell viability as described in "Data exclusions".
Randomization	The samples were not randomized, as there were no difference in terms of treatment/condition between the mice.
Blinding	Experiments were not blinded, as there were no difference in terms of treatment/condition between the mice.

Reporting for specific materials, systems and methods

We require information from authors about some types of materials, experimental systems and methods used in many studies. Here, indicate whether each material, system or method listed is relevant to your study. If you are not sure if a list item applies to your research, read the appropriate section before selecting a response.

Materials & experimental systems

n/a	Involved in the study	
☐	☒	Antibodies
☒	☐	Eukaryotic cell lines
☒	☐	Palaeontology and archaeology
☐	☒	Animals and other organisms
☒	☐	Clinical data
☒	☐	Dual use research of concern
☒	☐	Plants

Methods

n/a	Involved in the study	
☒	☐	ChIP-seq
☐	☒	Flow cytometry
☒	☐	MRI-based neuroimaging

Antibodies

Antibodies used

All antibodies used in this study are listed in Extended Data Table 2.

Validation

Validation of all primary antibodies could be found on the manufacturer's website, along with
relevant citations, and antibody profile :
Purified anti-mouse TCR β : https://www.biolegend.com/en-us/products/purified-anti-mouse-tcr-beta-chain-antibody-274
Purified anti-mouse CD117 : https://www.biolegend.com/en-us/products/purified-anti-mouse-cd117-c-kit-antibody-77
Purified anti-mouse CD49b : https://www.biolegend.com/en-us/products/purified-anti-mouse-cd49b-pan-nk-cells-antibody-235
Purified anti-mouse CD19 : https://www.biolegend.com/en-us/products/purified-anti-mouse-cd19-antibody-1532
Purified anti-mouse CD45 : https://www.biolegend.com/en-us/products/purified-anti-mouse-cd45-antibody-102
Purified anti-mouse CD4 : https://www.biolegend.com/en-us/products/purified-anti-mouse-cd4-antibody-484
Purified anti-mouse Ly-6G : https://www.biolegend.com/en-us/products/purified-anti-mouse-ly-6g-antibody-4767
Purified anti-mouse Ly-6C : https://www.biolegend.com/en-us/products/purified-anti-mouse-ly-6c-antibody-4894
Purified anti-mouse CD8a :https://www.biolegend.com/en-us/products/purified-anti-mouse-cd8a-antibody-157
Purified anti-mouse CD115 (CSF-1R) : https://www.biolegend.com/en-us/products/purified-anti-mouse-cd115-csf-1r-antibody-6214
Purified anti-mouse CD43 : https://www.biolegend.com/en-us/products/purified-anti-mouse-cd43-antibody-7589
Purified anti-mouse Ly-6A/E (Sca-1) : https://www.biolegend.com/en-us/products/purified-anti-mouse-ly-6a-e-sca-1-antibody-230
Purified anti-mouse I-A/I-E : https://www.biolegend.com/en-us/products/purified-anti-mouse-i-a-i-e-antibody-368
Purified anti-mouse/human CD11b : https://www.biolegend.com/en-us/products/purified-anti-mouse-human-cd11b-antibody-351

Primary conjugates of mass cytometry antibodies were prepared using the Maxpar antibody conjugation kit (Fluidigm Inc.) according to the manufacturer protocol (PRD002 Fluidigm Inc.) and optimal concentration was determined by titration according to the manufacturer protocol.

Animals and other research organisms

Policy information about studies involving animals; ARRIVE guidelines recommended for reporting animal research, and Sex and Gender in Research

Laboratory animals

The Collaborative Cross founder strain of the laboratory strains A/J, C57BL/6J, 129S1Sv/ImJ, NOD/ShiLtJ, NZO/H1LtJ, and wild-derived strains CAST/EiJ, PWK/PhJ, and WSB/EiJ were purchased from The Jackson Laboratory, delivered to the Systems Genetics Core Facility at The University of North Carolina, and sacrificed. Altogether, we profiled 23 founder mice aged 6- 8 weeks. The Collaborative Cross recombinant mice were both purchased from, handled, bred and sacrificed by the Systems Genetics Core Facility at The University of North Carolina. Altogether, we profiled 129 Collaborative Cross mice aged 8-14 weeks.
Mice handaling: mice were group-housed in GM500 Green Line individually ventilated caging (Tecniplast, Buguggiate, Italy) with 70 air exchanges per hour. The room was maintained between 70 and 74°F with 30 -70 % humidity and a 12-h light cycle.

Wild animals

The study did not include wild animals

Reporting on sex

All the mice were male

Field-collected samples

The study did not include samples collected from the field

Ethics oversight

All procedures involving animals were performed according to the Guide for the Care and Use of Laboratory Animals with prior approval by the Institutional Animal Care and Use Committee within the Association for Assessment and Accreditation of Laboratory Animal Care-accredited program at the UNC at Chapel Hill (Animal Welfare Assurance Number: A-3410-01)

Note that full information on the approval of the study protocol must also be provided in the manuscript.

Plants

Seed stocks | N/A

Novel plant genotypes | N/A

Authentication | N/A

Flow Cytometry

Plots

Confirm that:

☒ The axis labels state the marker and fluorochrome used (e.g. CD4-FITC).

☒ The axis scales are clearly visible. Include numbers along axes only for bottom left plot of group (a 'group' is an analysis of identical markers).

☒ All plots are contour plots with outliers or pseudocolor plots.

☒ A numerical value for number of cells or percentage (with statistics) is provided.

Methodology

Sample preparation	Cells from each sample were washed twice with Cell Staining Medium (Maxpar) and a total of three million cells were used for extracellular staining. Cells were resuspended in 500 µl containing 1:2000 rhodium DNA intercalator (Fluidigm) for 20 min of live/dead cells staining. Samples were washed with Cell Staining Medium and resuspended in a total of 100 µl metal tagged antibody mix for 1 hour, for cell surface marker staining. Cells were then fixed with 1.6% PFA (Sigma-Aldrich) in a total volume of 200 µl and stored at 4°C. Cells were centrifuged, PFA removed and iridium DNA intercalator staining was performed for 20 min at 1:2000 dilution in 500 µl volume to differentiate cells from debris. Finally, fixed samples were washed 3 times with deionised water immediately prior to data acquisition.
Instrument	CyTOF1 machine (DVS Sciences)
Software	CyTOF 1 acquisition software (v1)
Cell population abundance	Cell population abundances were defined by manual gating. The resulting frequencies are available in Extended Data Tables 4 and 6.
Gating strategy	Detailed gating strategy is shown in Supplementary Figure 1.

☒ Tick this box to confirm that a figure exemplifying the gating strategy is provided in the Supplementary Information.
